# Effect of Silicon and Continuous Annealing Process on the Microstructure, Mechanical Properties, and Hydrogen Embrittlement of DP1500 Steel

**DOI:** 10.3390/ma19010006

**Published:** 2025-12-19

**Authors:** Wei Li, Yu Tang, Boyu Cao, Yeqian Jiang, Yang Shen, Wei Li, Ke Zhang

**Affiliations:** 1 School of Materials and Chemistry, University of Shanghai for Science and Technology, Shanghai 200093, China; liwei700380@baosteel.com (W.L.); 243403063@st.usst.edu.cn (Y.T.); boyucao163@163.com (B.C.); jyq20220401@163.com (Y.J.); shenyang32100@163.com (Y.S.); liwei176@usst.edu.cn (W.L.); 2 Research Institute, Baoshan Iron & Steel Co., Ltd., Shanghai 201999, China

**Keywords:** dual-phase steel, silicon, continuous annealing process, retained austenite, mechanical properties, hydrogen embrittlement

## Abstract

Dual-phase (DP) steels are widely used in automotive structures due to their excellent strength–ductility balance. This study examines how silicon content and continuous annealing parameters affect the microstructure, mechanical properties, and hydrogen embrittlement (HE) behavior of DP1500 steel. Two steels, 05DP (0.5% Si) and 15DP (1.5% Si), were processed under annealing temperatures of 800–850 °C and over-aging temperatures of 240–300 °C. Higher annealing temperatures increased austenite formation and produced more martensite after cooling, leading to higher strength but reduced ductility at 850 °C due to martensite coarsening. Increasing the over-aging temperature coarsened carbides and reduced strength yet stabilized retained austenite and improved ductility through the TRIP effect. An increase in silicon content suppressed carbide precipitation, promoted carbon enrichment in austenite, refined the ferrite–martensite structure, and significantly enhanced both strength and elongation. Consequently, 15DP steel exhibited superior mechanical properties compared to 05DP steel, exhibiting 90–100 MPa higher tensile strength (+6.2–7.0%), 55–65 MPa higher yield strength (+5.3–6.2%), and 1.4–1.8 percentage points higher total elongation (+10–14%), resulting in a 16–20% increase in the strength–ductility balance (Rm × A). However, due to the relatively high hydrogen embrittlement susceptibility of fresh martensite formed either by the TRIP effect during deformation or after over-aging, 15DP steel did not exhibit substantially improved HE resistance despite its higher retained austenite fraction.

## 1. Introduction

Over the past few decades, Advanced High-Strength Steels (AHSSs) have been developed for manufacturing safe and energy-efficient automobiles [1,2]. One key innovation involves introducing martensite and austenite phases into the microstructure. Martensite significantly enhances mechanical strength, while austenite contributes to ductility, enabling the simultaneous achievement of high strength and plasticity [3,4]. A higher fraction and appropriate morphology (especially stable film-like) of retained austenite can deliver sustained TRIP effect, relaxing stress concentrations, delaying necking, and thus significantly improving uniform elongation and work-hardening without compromising strength [5,6]. Dual-phase (DP) steels, composed of hard martensite and soft ferrite, belong to the first generation of AHSSs commercialized in the early 1990s. Due to their combination of high tensile strength (up to 1.5 GPa), good ductility (e.g., 13%), and straightforward production processes [7,8], DP steels have attracted extensive attention in both industry and academia. Their adaptability to industrial processing and the demands of the automotive sector has led to widespread applications in structural components, reinforcements, and crash-resistant parts such as underbody crossmembers and bumpers [9,10,11].

Despite these advantages, hydrogen embrittlement (HE) remains a critical limitation for DP steels in automotive service. Unlike the hydrogen introduced during manufacturing processes such as pickling, plating, or welding, the more serious concern lies in unexpected hydrogen uptake during service exposure, which may result in premature failure of load-bearing components under dynamic stresses. Hydrogen accumulation at stress concentration sites (e.g., notches) significantly increases HE susceptibility and undermines the reliability of automotive structures [12,13,14]. Three primary factors influence hydrogen-induced delayed fracture, stress intensity, hydrogen concentration, and microstructure, among which microstructure plays the decisive role. The susceptibility of microstructural constituents to HE generally increases in the order: ferrite < lower bainite < tempered martensite < pearlite < martensite [15,16,17]. Retained austenite remains more complex: while it shows low diffusivity and high solubility for hydrogen, its stability determines whether it acts as a beneficial hydrogen trap and mitigates HE sensitivity or as a source of hydrogen release during martensitic transformation under stress. For instance, Figueroa et al. [18,19] demonstrated that reversed austenite in AeMet100 martensitic steel immobilized hydrogen and enhanced HE resistance compared with 300 M martensitic steel without retained austenite.

Alloying additions strongly influence the stability of retained austenite. Silicon, in particular, is widely recognized as an effective ferrite stabilizer that suppresses carbide precipitation, promotes carbon enrichment in austenite, and enhances solid-solution strengthening at relatively low cost [20,21]. Silicon is thermodynamically considered a ferrite stabilizer that suppresses carbide precipitation and thereby promotes carbon enrichment in austenite. Consequently, Si indirectly enhances the stability of retained austenite and improves the balance between strength and ductility. These findings suggest that Si is not only a solid-solution strengthening element but also a key factor influencing the morphology and stability of retained austenite, thereby modifying hydrogen trapping characteristics and hydrogen embrittlement susceptibility in DP steels. Saleh et al. [22] showed that Si additions up to ~1.5% in DP steel improve hardenability and toughness when combined with optimized heat treatment. Similarly, Davies and Thomas [23], as well as Fonstein and Hironaka et al. [24,25], reported that increasing Si content can achieve an improved balance of strength and ductility, with an optimum level around 2%. In addition, higher Si levels also increase the work hardening rate, which may influence crack resistance during deformation [26,27]. These findings suggest that Si is not only a strengthening element but also a key factor affecting the morphology and stability of retained austenite, thereby altering the hydrogen trapping characteristics and HE susceptibility of DP steels. Nevertheless, previous studies have shown that Si significantly influences the microstructural evolution and mechanical behavior of DP steels by suppressing carbide precipitation and stabilizing retained austenite [28]. However, despite extensive research on the mechanical strengthening mechanisms of Si, the quantitative relationship between Si-induced microstructural changes and hydrogen trapping behavior has not been fully clarified. In particular, the influence of Si on the hydrogen trapping energy, distribution of trap sites, and their correlation with the stability of retained austenite remains insufficiently understood.

In recent years, the influence of silicon on embrittlement susceptibility of continuously annealed dual-phase steels has attracted considerable attention. Kalashami and co-workers [29,30] systematically revealed that increasing silicon content from 0.7 wt.% to 1.8 wt.% during intercritical annealing of galvanized DP steels promotes ferrite coarsening, deeper surface decarburization, and significantly higher density of internal oxides. These microstructural heterogeneities provide preferential nucleation sites and fast-diffusion paths for embrittling species, dramatically aggravating liquid metal embrittlement (LME) cracking during resistance spot welding—a mechanism closely analogous to hydrogen embrittlement. Consequently, although higher silicon stabilizes retained austenite and improves strength–ductility balance, the resulting stressed phase interfaces and oxide-related traps simultaneously act as detrimental hydrogen accumulation sites, making it difficult to substantially enhance hydrogen embrittlement resistance solely by silicon addition.

Therefore, this work aims to fill this knowledge gap by systematically investigating the effect of Si content (0.5 wt.% and 1.5 wt.%) on the microstructure and hydrogen embrittlement (HE) resistance of DP1500 steel. By combining XRD, EBSD, and TEM analyses with slow strain rate testing (SSRT), this study provides new insights into how Si-tailored microstructures influence hydrogen trapping mechanisms and improve the balance between strength, ductility, and HE resistance.

## 2. Materials and Experimental Procedure

### 2.1. Experimental Materials

The experimental steels were laboratory researched DP1500 high-strength steel with a thickness of 1.2 mm. To investigate the influence of Si content on the microstructure and properties of DP steel, two composition systems with different Si contents (0.5% and 1.5%) were designed during this study. The experimental steels were designated as 05DP steel and 15DP steel, respectively. The chemical compositions of the experimental steels are shown in Table 1.

### 2.2. Heat Treatment Process

**Figure 1:**
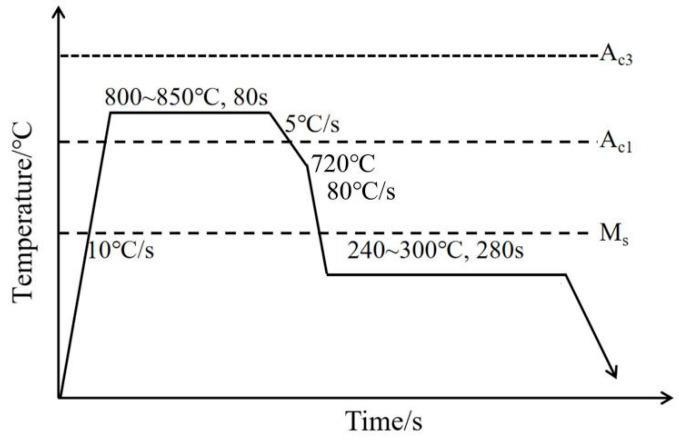
Heat treatment process of cold rolled DP1500 steel.

In this experiment, the phase transformation temperatures of two DP steels with different Si contents were determined using a dilatometer (Gleeble 3500-GTC, DSI, Poestenkill, NY, USA), as shown in Table 2. Figure 1 illustrates the continuous annealing process for 05DP and 15DP steels. The intercritical annealing temperatures were set to 800 °C, 825 °C, and 850 °C, with a holding time of 80 s. Subsequently, slowly cool down to 720 °C, then quickly cool down to the over-aging temperature of 240 °C, 270 °C, and 300 °C, and maintain this temperature for 280 s. To achieve the required cooling rate, compressed air (adjustable pressure/volume) is directed onto both surfaces of the steel plate via the cooling system. Thermocouples measure the plate’s surface temperature, recording temperature changes over a defined period to ultimately control cooling within 80 °C/s.

### 2.3. Microstructure Characterization

Metallographic specimens were prepared through grinding and polishing, then etched with a 4.0 vol% nitric acid alcohol solution for 10 s. The microstructural analysis was conducted with an FEI QUANTA 450 scanning electron microscope (SEM, FEI, Hillsboro, OR, USA). Microstructural features and precipitates were further examined using a Tecnai G2 F20 transmission electron microscope (TEM, FEI, Hillsboro, OR, USA). X-ray diffraction (XRD) analysis was performed using a Bruker D8 (Burker, Berlin, Germany) Advance diffractometer, with a scanning angle range of 35° to 90°. The retained austenite volume fraction V_γ_ was calculated using the standard integrated intensity ratio method [31]:

(1) $$V_{\gamma }=\frac{\sum \left(I_{\gamma (hkl)}/R_{\gamma (hkl)}\right)}{\sum (I_{\gamma (hkl)}/R_{\gamma (hkl)})+\sum \left(I_{\alpha (hkl)}/R_{\gamma (hkl)}\right)}$$

where I_phase(hkl)_ represents the integrated intensity of each reflection, and R_phase(hkl)_ is the theoretical relative intensity factor including multiplicity, Lorentz–polarization correction, and structure factor. For EBSD testing, the accelerating voltage was set to 20 kV, and the scan step size was set to 0.05 μm.

### 2.4. Mechanical Performance Test

**Figure 2:**
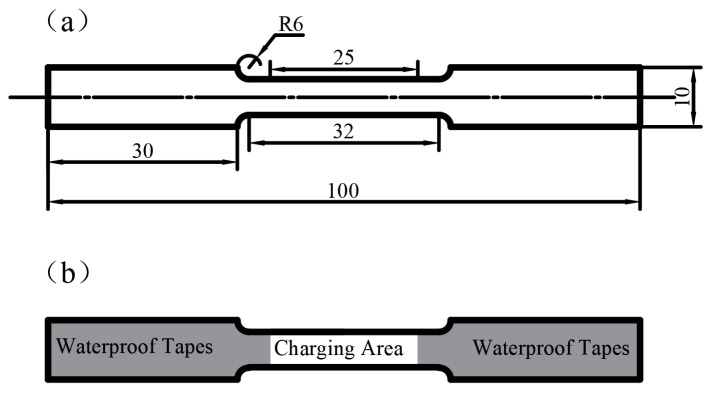
(**a**) Geometry of tension specimens, all dimensions in millimeter. Schematic diagram of the (**b**) SSRT specimen subjected to hydrogen charging [32].

Flat tensile samples, as shown in Figure 2, were prepared to the gage length of 25 mm, a width of 6 mm, and thickness of 1.2 mm. Tensile tests were performed on a UTM4304 material tensile testing machine (Suns, Zhuhai, China) at a strain rate of 0.5 mm/min at room temperature. Three tests were conducted for each sample group, and the average value was calculated.

To quantitatively verify the occurrence and contribution of the transformation-induced plasticity (TRIP) effect during tensile deformation, interrupted tensile tests combined with ex situ XRD measurements were carried out on both 05DP and 15DP steels processed at 850 °C annealing +240 °C over-aging (the condition exhibiting optimum comprehensive properties). Standard flat tensile specimens (gauge length 25 mm, width 6 mm) were stretched to engineering strains of 0%, 5%, and immediately before necking, respectively. After unloading, small specimens were cut from the central uniform deformation zone of the gauge section, mechanically ground, polished, and lightly electropolished to remove the surface deformed layer. XRD measurements were performed using the same Bruker D8 Advance diffractometer (Co Kα radiation, 35–90°), and the volume fraction of retained austenite (Vγ) was calculated by the integrated intensity ratio method described in Section 2.3. Three parallel samples were tested for each strain level to ensure reproducibility.

### 2.5. Hydrogen Embrittlement Sensitivity Test

The experimental steel plates were wire-cut into hydrogen permeation specimens, with dimensions of 50 mm × 20 mm × 1.2 mm. After grinding, the specimens were placed in an electrolytic cell. Under the operation of a constant current power supply, oxidation currents were generated to obtain hydrogen permeation curves. Hydrogen permeability (J∞L), hydrogen trap (N_T_), effective hydrogen diffusion coefficient (D_eff_), and apparent hydrogen concentration (C_app_) were calculated using the following formulas.

(2) $$I_{\infty }L=\frac{I_{\infty }\times L}{F\times A}$$

(3) $$D_{eff}=\frac{L^{2}}{6t_{L}}$$

(4) $$C_{app}=\frac{J_{\infty }L}{D_{eff}}$$

(5) $$N_{T}=\frac{N_{A}\times C_{app}}{3}\left(\frac{D_{1}}{D_{eff}}-1\right)$$

Here, A is the effective hydrogen-charging area (78.5 mm^2^ in this experiment), D_1_ is the lattice diffusion coefficient of hydrogen (1.28 × 10^−4^ cm^2^ s^−1^), F is the Faraday constant (96485C mol^−1^), and N_A_ is Avogadro’s number (6.02 × 10^23^ mol^−1^).

After pre-hydrogen charging treatment (0.5 mol/L H_2_SO_4_ + 1g/L CH_4_N_2_S), SSRT tests were conducted on the samples at a rate of 0.015 mm/min. This experiment was repeated three times under different hydrogen charging times and current densities. The hydrogen charging conditions were as follows: the fixed charging time was 1 min, and the current density increases from 1 mA/cm^2^, 2 mA/cm^2^ to 15 mA/cm^2^. When the fixed current density is 2 mA/cm^2^, the time was 5 and 10 min. The HE susceptibility index (El_loss_, Elongation loss) was calculated using Formula (6) to determine the impact of hydrogen charging conditions on the HE sensitivity of both DP steels.

(6) $${El}_{Loss}=\frac{{El}_{Uncharged}-{El}_{charged}}{{El}_{Uncharged}}\times 100\%$$

The experimental steel plates were machined by wire cutting into hydrogen thermal desorption spectrometer (TDS) specimens of 20 mm × 5 mm × 1.2 mm. After polishing and being charged with hydrogen (0.2 mol NaOH + 2 g/L CH_4_N_2_S, charging 24 h), the contents of diffusible hydrogen and non-diffusible hydrogen in the test steels were measured using the HTDS-002 Hydrogen TDS (RDEC, Tokyo, Japan). The heating temperature range set for the experiment was 25~850 °C, with a heating rate of 100 °C/h. The activation energy (E_α_) associated with the hydrogen traps corresponding to the peaks observed in the TDS spectra was determined using the approximate Formula (7) proposed by P.A. Redhead [27]. In this expression, β is the heating rate (K/s), T_p_ is the desorption peak temperature (K) in TDS, R is the gas constant (8.314 J·mol^−1^·K^−1^), and v is the attempt frequency (taken as 10^13^ s^−1^ in this study).

(7) $$E_{\alpha }=RT_{p}\left[\ln\left(\frac{vT_{p}}{\beta }\right)-3.64\right]$$

## 3. Experimental Results

### 3.1. Effect of Annealing Temperature on the Microstructure of DP1500 Steels

**Figure 3:**
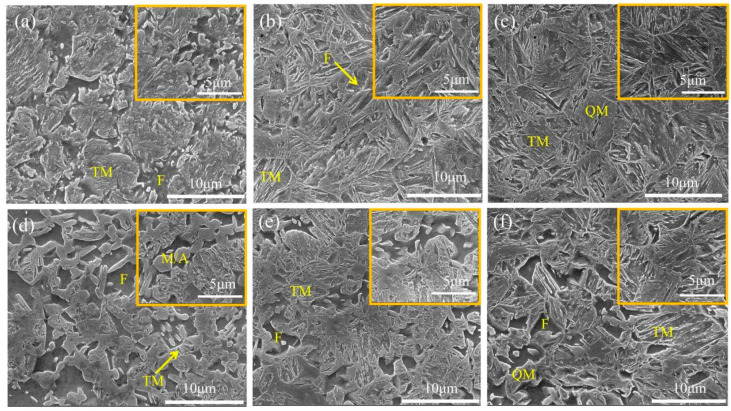
SEM images of 05DP steel and 15DP steel annealed at (**a**) and (**d**) 800 °C, (**b**) and (**e**) 825 °C, (**c**) and (**f**) 850 °C, followed by over-aging at 240 °C, respectively. Note: F is Ferrite; QM is Quenched Martensite; TM is Tempered Martensite; M/A is martensite/austinite island.

Figure 3 shows the SEM images of 05DP and 15DP steels annealed at different temperatures and overaged at 240 °C. It can be seen from Figure 3 that both experimental steels mainly consist of martensite as the matrix, with ferrite uniformly dispersed within it. This is exactly the opposite of the traditional low-intensity DP steel. Its microstructure consists of ferrite as the matrix, with island-shaped martensite distributed on the matrix. When 05DP steel is annealed at 800 °C (Figure 3a), due to the relatively low annealing temperature, less austenite is generated in the two-phase region, resulting in relatively less martensite transformed from the austenite. After tempering at 240 °C, the content of blocky tempered martensite, transformed from some quenched martensite, is also relatively low. As the annealing temperature continues to rise, especially at 850 °C (Figure 3c), the volume fraction of austenite generated in the two-phase region gradually increases, but the carbon content of austenite relatively decreases. This will reduce the stability of austenite. During the subsequent cooling process, the rapid cooling rate causes a large amount of austenite to transform into quenched martensite. In the subsequent over-aging isothermal stage, some martensite undergoes tempering. Compared with 05DP steel, Figure 3d shows that in 15DP steel annealed at 800 °C, the block-like martensite and M/A islands are distributed in the ferrite matrix. It can be seen that as the Si content increases, the content of ferrite significantly increases during low-temperature annealing, but as the annealing temperature rises, its content also gradually decreases. This indicates that silicon promotes carbon enrichment in austenite, inhibits cementite formation, and purifies ferrite [29]. With the increase in annealing temperature, the content of M/A islands decreases, the volume fraction of the blocky martensite increases, and the martensite plates become significantly larger. Meanwhile, the refinement of ferrite grains and the homogenization of the structure have been enhanced.

**Figure 4:**
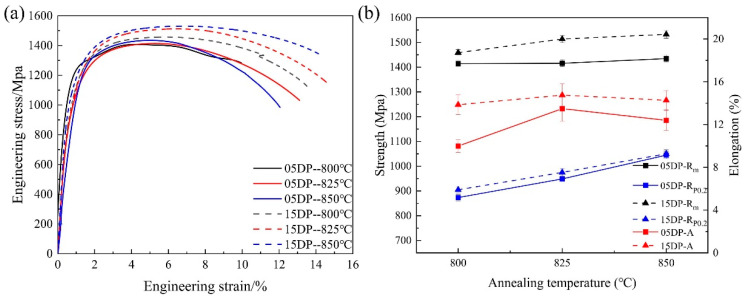
Engineering stress–strain curves (**a**) and the mechanical properties (**b**) for 05DP steel and 15DP steel annealed at different temperatures and overaged at 240 °C.

Figure 4 shows the engineering stress–strain curves and the variation in the mechanical properties of 05DP steel and 15DP steel at different annealing temperatures and overaged at 240 °C. As can be seen from Figure 4a, both the experimental steels exhibit continuous yielding behavior without an obvious physical yield point and yield elongation zone when annealed at different temperatures. This is a typical characteristic of high-strength steels with martensite as the matrix. This is because the volume expansion caused by the martensitic transformation during rapid cooling exerts compressive stress on the surrounding structure, equivalent to pre-deforming the surrounding structure, resulting in weakened pinning effect of atoms during tensile testing, manifested as no obvious yield plateau [30]. Figure 4b shows the variations in the yield strength (R_p_0.2), ultimate tensile strength (R_m_), and total elongation (A) for all the specimens annealed at different temperatures from 800 °C to 850 °C. The results indicate that all the specimens subjected to continuous annealing process exhibit high strength and elongation. With the increase in annealing temperature, both R_p_0.2 and R_m_ increase, while A first increases and then decreases. For example, when the annealing temperature was 825 °C, 05DP steel possess the best comprehensive mechanical properties: R_p_0.2 was 949.9 MPa, R_m_ was 1413.6 MPa, and A was 13.11%. Compared with 05DP steel, under the same continuous annealing process conditions, 15DP steel exhibits more superior comprehensive mechanical properties: R_p_0.2 was 974.7 MPa, R_m_ was 1512.1 MPa, and A was 14.58%. It can be concluded that the Si inhibits cementite precipitation, enriches carbon in austenite, forms high-carbon martensite after quenching, and simultaneously enhances the strength of the ferrite matrix through solid-solution strengthening.

### 3.2. Effect of Over-Aging Temperature on DP1500 Steels

**Figure 5:**
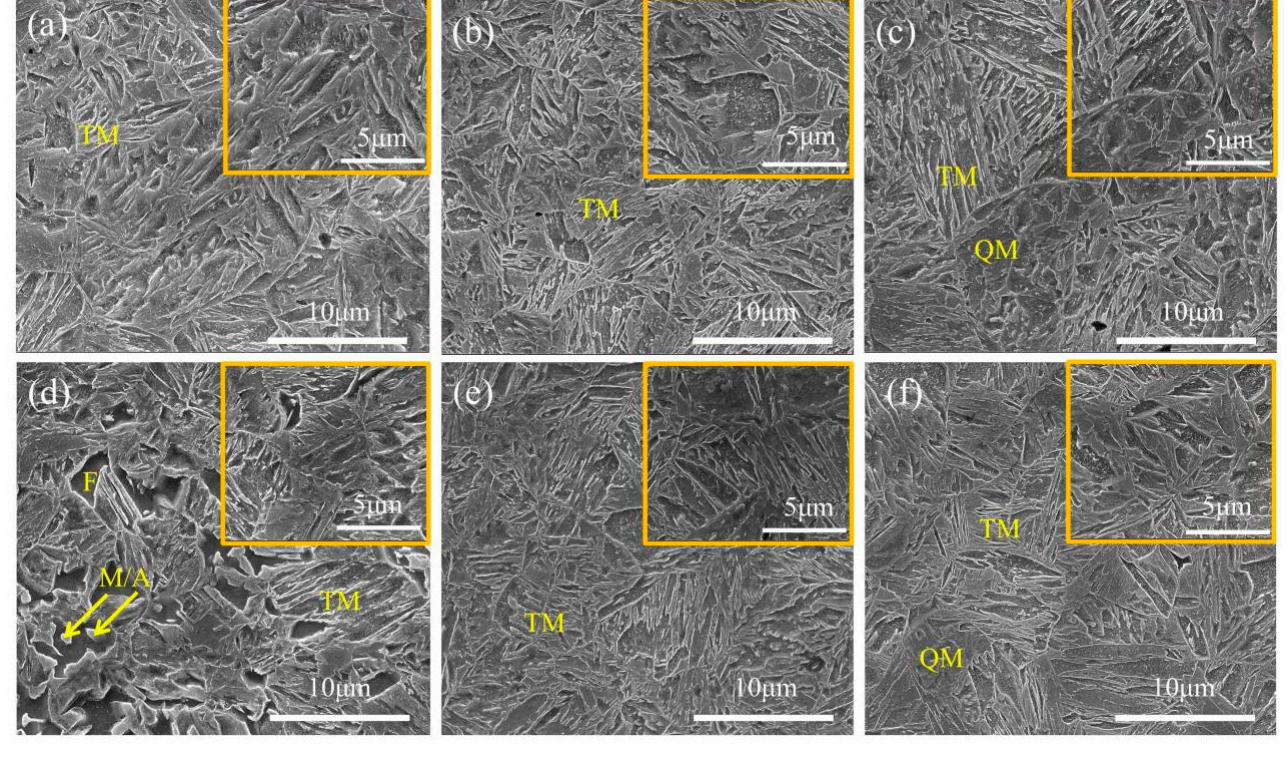
SEM images of 05DP steel and 15DP steel annealed at 850 °C and over-aging at (**a**) and (**d**) 240 °C, (**b**) and (**e**) 270 °C, and (**c**) and (**f**) 300 °C, respectively. Note: F is Ferrite; QM is Quenched Martensite; TM is Tempered Martensite; M/A is marten-site/austinite island.

Figure 5 shows the SEM images of 05DP steel and 15DP steel annealed at 850 °C and overaged at different temperatures from 240 °C to 300 °C. It is clearly shown that all the specimens subjected to the continuous annealing process have identical multiphase microstructure: polygonal ferrite, quenched martensite, and tempered martensite. When the over-aging temperature is relatively low, such as 240 °C (Figure 5a), the microstructure mainly consists of less ferrite and a large amount of tempered martensite. During the rapid cooling process before over-aging treatment, most of the austenite transforms into martensite. This portion of martensite undergoes an annealing transformation during isothermal aging and becomes tempered martensite. When the over-aging temperature rises to 300 °C, which is further away from M_f_, less transformed martensite is obtained during the rapid cooling process before over-aging treatment, and consequently, the proportion of tempered martensite also decreases. This also means that a larger amount of austenite is retained. During the subsequent quenching process after over-aging treatment, some of the unstable austenite will further transform into fresh martensite. Meanwhile, as the over-aging temperature increases, the tempered martensite gradually decomposes. Its typical characteristic of lamellar structure is still retained, but carbides begin to precipitate. Furthermore, by comparing Figure 5c,f, it can be observed that the decomposition degree of the tempered martensite in 15DP steel with high Si content is less than that in 05DP steel.

**Figure 6:**
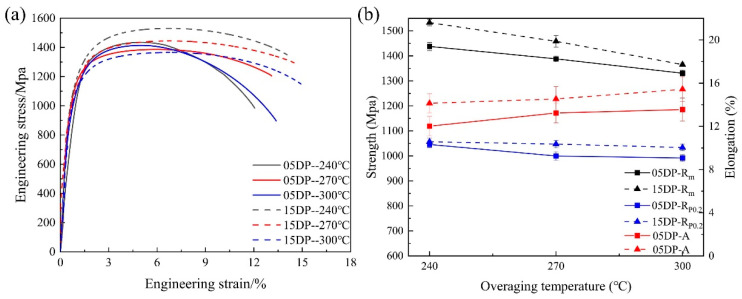
(**a**) Engineering stress–strain curves and (**b**) mechanical properties of 05DP steel and 15DP steel annealed at 850 °C and overaged at different temperatures.

Figure 6a shows the engineering stress–strain curves of 05DP steel and 15DP steel annealed at 850 °C and overaged at different temperatures from 240 °C to 300 °C, and the corresponding variation in the mechanical properties are plotted in Figure 6b. The results indicate that R_m_ of both the experimental steels gradually decrease with the increase of over-aging temperatures, but the reduction in R_p0.2_ is relatively small. When the over-aging temperature increases from 240 °C to 300 °C, R_m_ of 05DP steel decreases from 1434.9 MPa to 1327.2 MPa, and R_m_ of 15DP steel decreases from 1530.4 MPa to 1365.5 MPa; R_p0.2_ of 05DP steel decreases from 1043.6 MPa to 990.7 MPa, and R_p0.2_ of 15DP steel decreases from 1055.5 MPa to 1031.6 MPa. The elongation of 05DP steel and 15DP steel gradually increase, which is 13.38% and 15.11% at the overaged temperature of 300 °C, respectively. It is worth noting that, the mechanical properties of 15DP steel are superior to those of 05DP steel at the same over-aging temperature. Based on the above mechanical properties results, in this paper, the samples that were annealed at 850 °C and overaged at 240 °C were selected for further characterization and for the following evaluation of HE sensitivity.

### 3.3. Microstructural Characterization of DP1500 Steels

**Figure 7:**
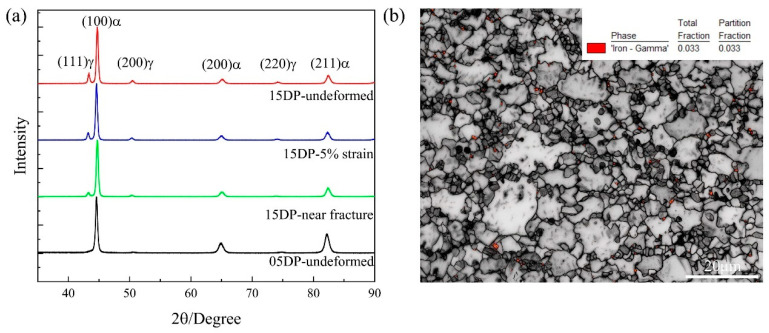
(**a**) XRD spectra of 05DP steel and 15DP steel; (**b**) EBSD map of 15DP steel by combining band contrast map and phase map.

Figure 7a shows the XRD spectra of 05DP steel and 15DP steel annealed at 850 °C and overaged at 240 °C. It can be seen that the samples treated by the continue annealing process consist of both martensite and retained austenite. Compared with the 05DP sample, the austenite peaks intensity of (111)_γ_, (200)_γ_, and (220)_γ_ in the 15DP sample were significantly greater. The retained austenite fraction was quantified using the standard integrated intensity ratio (IIR) method. This method is widely adopted for phase quantification in multiphase steels, and numerous studies have confirmed that it can reliably achieve accuracy up to two decimal places when proper peak fitting and background correction are applied [30]. Therefore, the values reported here reflect the typical precision attainable by the IIR method. To further verify the content and distribution location of retained austenite, Figure 7b shows the EBSD map by combining band contrast map and phase map of the 15DP specimen. In Figure 7b, the red regions correspond to austenite of an fcc lattice. It can be clearly observed that the retained austenite with blocky equiaxed morphology is situated within ferrite grains and at the original austenite grain boundaries. The volume fraction of retained austenite identified by EBSD image is 3.3%, which is far below the result calculated by XRD spectra. This is mainly due to the fact that the retained austenite distributed between the lath martensite is difficult to be detected, and during the preparation of EBSD samples, stress-induced martensitic phase transformation occurs [33].

**Figure 8:**
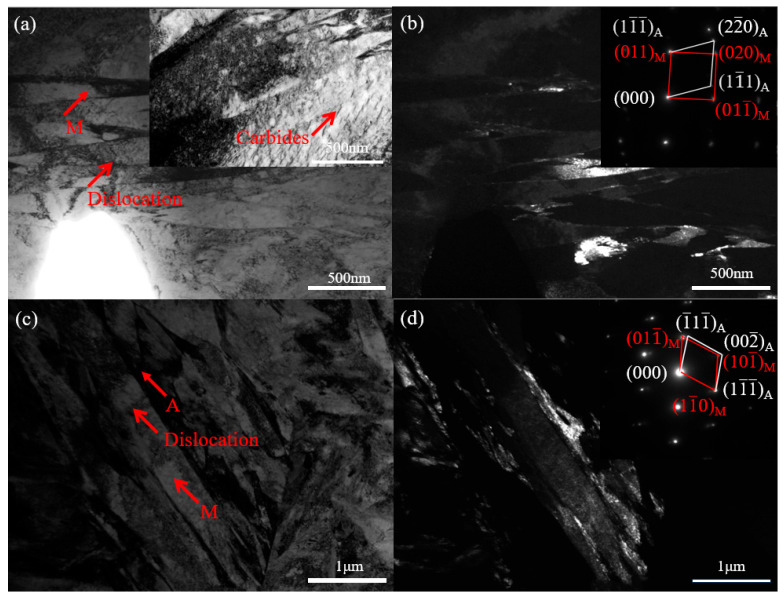
Bright field (**left**) and corresponding dark field (**right**) images of the 05DP (**a**,**b**) and 15DP (**c**,**d**) specimens, with the corresponding SAED patterns inserted in (**b**) and (**d**), respectively.

To better observe the effect of silicon on the microstructure of 05DP steel and 15DP steel, TEM was used to characterize the experimental steels with different silicon contents. Figure 8 shows the bright field image of martensite and the dark field image of retained austenite, as well as the corresponding selected area electron diffraction (SAED) patterns of 05DP steel and 15DP steel. The investigated 05DP and 15DP steels exhibited the representative microstructure, both of which consist of the lath martensite matrix and the film-like retained austenite distributed within the lath martensite. The orientation relationship (OR) [34,35] between the lath martensite and the retained austenite is revealed by SAED patterns as [0$\bar{1}$1]α//[$\bar{1}$2$\bar{1}$]γ, (011)α//(1$\bar{1}\bar{1}$)γ (N-W relationship), as shown in Figure 8b, and [$\bar{1}\bar{1}\bar{1}$]α//[$\bar{1}$0$\bar{1}$]γ, ($1\bar{1}$0)α//(1$\bar{1}\bar{1}$)γ (K-S relationship), as shown in Figure 8d. Meanwhile, it is observed that the average thickness of the lamellar retained austenite in the 15DP specimen is about 70 nm, which is significantly wider compared with that of the 05DP specimen with lower Si content. Moreover, the volume fraction of retained austenite in 15DP specimen is significantly higher than that in 05DP specimen. Additionally, TEM image inserted in Figure 8a clearly shows that there are a large number of transition type ε carbides with a laminar form. The ε-carbides were identified as independent hcp ε-Fe_2_._4_C transition carbides based on our previous research on ε-carbides [36].

### 3.4. Hydrogen Embrittlement Sensitivity of DP1500 Steels

**Figure 9:**
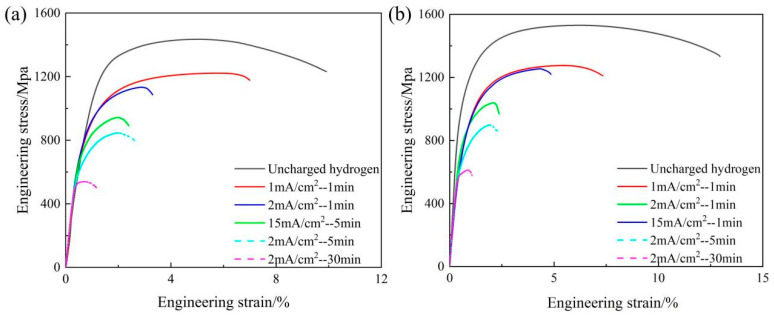
SSRT curves of 05DP sample (**a**) and 15DP sample (**b**), cathodically hydrogen charged at different charging times and current densities.

SSRT curves of 05DP steel and 15DP steel in the condition of hydrogen charging with different charging times and current densities are presented in Figure 9, and the corresponding mechanical properties are listed in Table 3. It can be observed that, compared with the as-received samples [37], the mechanical properties of the hydrogen charging samples all show a decreasing trend with the increase in charging times and current density. When the charging time was 1 min and current density was 1 mA/cm^2^, R_m_ of the 05DP sample rapidly dropped to 1215.87 MPa, and El_loss_ was 35.4%. Accordingly, the 15DP sample also rapidly dropped to 1274.48 MPa, and El_loss_ was 19.5%. It is worth noting that, as the hydrogen charging time and current density increase, the 15DP samples show a smaller El_loss_ compared to the 05DP samples.

**Figure 10:**
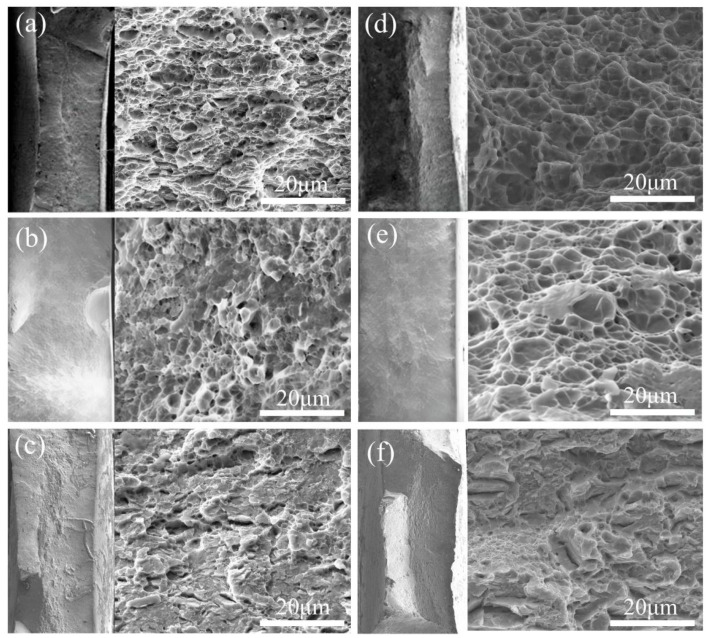
SEM fracture photographs of 05DP sample (**a**–**c**) and 15DP sample (**d**–**f**) at different hydrogen charging current densities. (**a**,**d**) Uncharged hydrogen; (**b**,**e**) 2 mA/cm^2^, 1 min; (**c**,**f**) 15 mA/cm^2^, 1 min.

Figure 10a,d show the tensile fracture photographs of 05DP and 15DP steels without hydrogen charging. Both materials exhibit typical ductile fracture characterized by dimples. For 05DP steel, the fracture surface is dominated by fine and uniformly distributed dimples, while 15DP steel shows larger and deeper dimples, indicating superior plasticity and toughness. After hydrogen charging, the fracture photographs of 05DP steel (Figure 10b,c) changes significantly. The number of dimples decreases, and some regions exhibit flat surfaces and quasi-cleavage features, suggesting a stronger tendency toward brittle fracture, indicating a higher susceptibility to HE. In contrast, 15DP steel at a charging current density of 2 mA/cm^2^ (Figure 10e) still shows predominantly dimpled fracture, although the dimples become coarser. The overall ductility is largely preserved, demonstrating a stronger resistance to HE. However, when the charging current density is increased to 15 mA/cm^2^ (Figure 10f), the fracture surface exhibits obvious brittle features, with a reduced number of dimples and the presence of cleavage facets. This indicates that even in high-Si 15DP steel, excessive hydrogen can still induce a transition from ductile to brittle fracture, eventually leading to hydrogen-induced failure.

## 4. Discussion

### 4.1. Effect of Si Content on Microstructure and Mechanical Properties of DP1500 Steels

As shown in Figure 3, with the increase in annealing temperature, the content of austenite in the two-phase region increases, but the carbon content in the austenite decreases, indicating that the austenite becomes unstable. During the subsequent rapid cooling process, the amount of quenched martensite increases. In the subsequent over-aging isothermal stage, the quenched martensite will undergo tempering to become tempered martensite. Therefore, as the annealing temperature increases, both R_p_0.2 and R_m_ of both 05DP steel and 15DP steel increase (Figure 4). However, the elongation shows a trend of increasing first and then decreasing. This is mainly attributed to the fact that as the annealing temperature increases, the ferrite content continuously decreases, while the martensite content increases and the lamellar structure becomes coarser simultaneously [28].

When the over-aging temperature is relatively low, the microstructure of 05DP steel consists of a small amount of ferrite and a large amount of tempered martensite. However, at this point, there is a significant amount of ferrite and a small amount of M/A islands present in the 15DP steel. As the over-aging temperature increases, the proportion of austenite transforming into martensite before isothermal over-aging decreases, thus the proportion of tempered martensite also decreases accordingly. Meanwhile, a certain amount of residual austenite is retained. During the subsequent quenching process after over-aging, some of the retained austenite will transform into secondary quenched martensite or fresh martensite. However, when the over-aging temperature reaches a certain level, for instance, 300 °C, the tempered martensite gradually decomposes and carbides begin to precipitate. Therefore, R_m_ of both the 05DP steel and 15DP steel gradually decreases with the increase of over-aging temperatures, but R_p_0.2 slightly decreases. Meanwhile, the elongation of 05DP and 15DP steels gradually increases.

Just as the aforementioned experimental results indicate that the mechanical properties of 15DP steel are superior to those of 05DP steel under the same continuous annealing process. The difference between 15DP steel and 05DP steel lies in that 15DP steel has a higher content of Si (1.5 wt %). An increase in Si content will cause carbon to concentrate in the austenite, thereby enhancing the hardenability and stability of the austenite. Instead, austenite transforms into proeutectoid ferrite during subsequent cooling to room temperature, thereby increasing the ferrite volume fraction [38]. According to the characterization results of XRD (Figure 7a), EBSD (Figure 7b), and TEM (Figure 8c), 15DP steel contains a considerable amount of retained austenite distributed between lamellar martensite laths, inside ferrite grains, and along the prior austenite grain boundaries. It should be noted that the quantitative measurement of retained austenite was obtained by XRD rather than by SEM analysis. The XRD results indicate that the retained austenite fraction increases from 4.65% in 05DP steel to 7.32% in 15DP steel, corresponding to an increment of approximately 2.67%. This confirms that increasing Si content slightly enhances the stability and amount of retained austenite within the ferrite–martensite matrix. At present, in the microstructure design of multiphase steels, the utilization of the DARA (dislocation absorption by the retained austenite) effect, TRIP (transformation-induced plasticity) effect, and BCP (block crack propagation) effect of retained austenite to simultaneously enhance the strength and plasticity of high-strength steels is being widely adopted [39,40]. Furthermore, an increase in Si content will enhance the ferrite content in 15DP steel, while inhibiting the formation of cementite, purifying the ferrite matrix, and improving the morphology and distribution of martensite [41]. As indicated in Figure 8a, a large amount of hcp-ε carbide precipitation was observed in the 05DP steel with low Si content. During the tensile process, as the stress increases, the soft ferrite and austenite phases will first undergo deformation, which will result in an increase in the dislocation density of both phases. The retained austenite with a face-centered cubic structure can accommodate a considerable number of dislocations during deformation because it has a large number of slip systems. Meanwhile, the retained austenite and martensite usually bear a coherent or semi-coherent interface. These properties can effectively reduce the resistance to the movement of dislocations from the martensite across the phase interface to the retained austenite. As the stress continues to increase, when the dislocation density in the austenite reaches the critical value, a strain-induced martensitic transformation will occur, that is, stress concentration will be released or redistributed through the TRIP effect. In summary, the superior strength–ductility combination of 15DP steel primarily arises from Si-induced synergistic effects: refinement of ferrite and martensite structure, increased volume fraction and enhanced stability of retained austenite, and sustained TRIP effect during deformation. These factors collectively promote progressive strain hardening and coordinated deformation among phases, leading to simultaneous improvements in strength (~80–100 MPa) and elongation (~1–2%) compared to 05DP steel.

The superior strength–ductility combination of 15DP steel is attributed to both ferrite purification/martensite refinement and a more pronounced TRIP effect during deformation. To confirm the occurrence of the TRIP effect experimentally, interrupted tensile tests combined with quantitative XRD analysis were performed on the 15DP steel processed at 850 °C annealing +240 °C over-aging. As shown in Figure 7a, the retained austenite fraction in 15DP steel decreases progressively from 7.32% in the undeformed state to 6.81% after 5% engineering strain, and further to 5.17% immediately before fracture. This continuous reduction in retained austenite during tensile deformation provides direct experimental evidence of strain-induced martensitic transformation (i.e., the TRIP effect) in 15DP steel. Owing to the higher initial volume fraction and appropriate mechanical stability of the film-like retained austenite (promoted by the higher Si content), the TRIP effect in 15DP steel is stronger and more sustained than in 05DP steel, which effectively extends the work-hardening stage, delays necking, and contributes to the observed improvement of ~1.4–1.8 percentage points in total elongation.

### 4.2. Effect of Si Content on Hydrogen Embrittlement Susceptibility of DP1500 Steels

**Figure 11:**
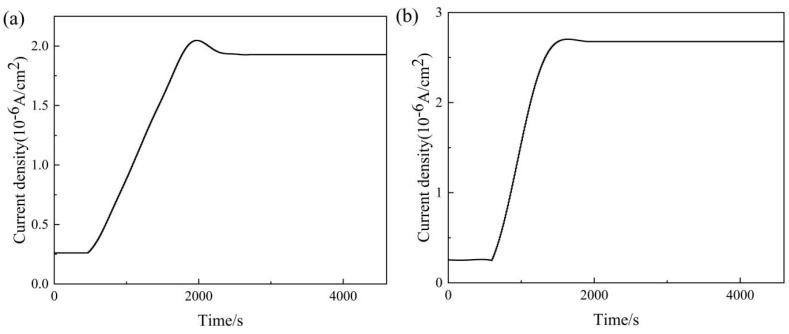
Hydrogen permeation curve: (**a**) 05DP steel; (**b**) 15DP steel.

By comparing the SSRT curves of the two experimental steels, it was found that with increasing hydrogen charging time and current density, El_loss_ of the experimental steels increases significantly. Furthermore, the results show that the 15DP steel with a higher content of ferrite phase and austenite phase does not exhibit a more outstanding resistance to HE. This is contrary to the view held by some researchers [42]. Therefore, the hydrogen permeation curves for 05DP and 15DP steels are presented in Figure 11, and the calculated values of the J∞L, C_app_, D_eff_, and N_T_ are listed in Table 4. It was found that the 15DP steel has the lower D_eff_ and the higher N_T_. The presence of more austenite in the organization, due to its lower hydrogen diffusion coefficient and higher hydrogen solubility, results in higher resistance to HE. Fractographic observations therefore reveal that 15DP steel under hydrogen charging predominantly fails through microvoid coalescence, whereas 05DP steel exhibits more quasi-cleavage features and secondary cracks. This indicates that 05DP steel is more susceptible to HE under identical charging conditions. The underlying difference is closely related to the influence of Si content on microstructure and hydrogen behavior. A higher Si content not only enhances the solid-solution strengthening of the matrix, suppresses carbide precipitation, and promotes a more uniform phase distribution, but may also modify the nature and distribution of hydrogen traps.

**Figure 12:**
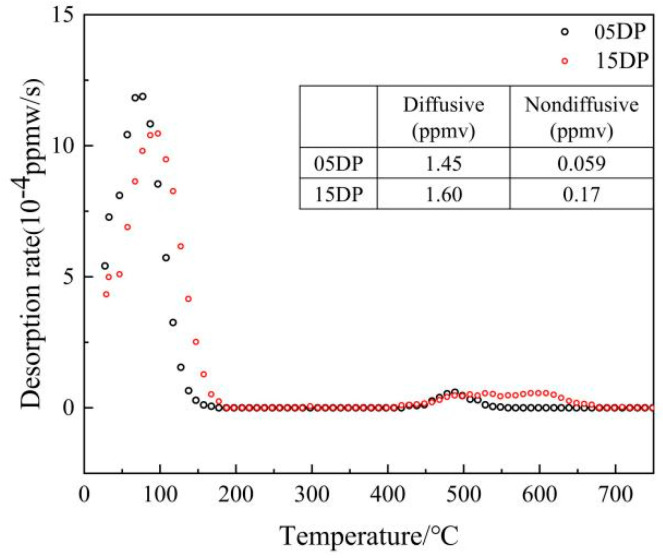
Typical hydrogen desorption spectra of 05DP and 15DP steels. The inserted table is a summary of the hydrogen content.

Moreover, typical TDS curves of 05DP and 15DP steels after the same charging conditions are shown in Figure 12, and the corresponding diffusible and non-diffusible hydrogen contents are inserted in the figure. It was shown that two pronounced desorption peaks induced by hydrogen charging: a strong peak appears at approximately 90~120 °C, and another weaker peak appears at 450~650 °C. It is generally believed that the low-temperature peak corresponds to the reversible hydrogen trap, while the high-temperature peak corresponds to the irreversible hydrogen trap [43]. The TDS results indicate that the diffusible hydrogen content and the non-diffusible hydrogen content in 15DP steel are both higher than those in 05DP steel. In addition, a distinct shoulder can be clearly observed at the beginning of the spectrum of 15DP steel. Boot et al. [44] proposed that the first shoulder in the TDS spectrum is related to hydrogen trapped by dislocations. This indicates that deformation-induced defects such as dislocations, represented by the increase in GNDs, can weakly trap hydrogen. In 15DP steel, the increased Si content promotes the enrichment of C in martensite, thereby increasing dislocation density. Meanwhile, the TDS peaks of 15DP steel shift slightly to higher temperatures, which may be associated with the reduction in effective hydrogen diffusivity caused by the increased Si content [45] determined using the Redhead equation. In 05DP steel, the low-temperature peak corresponds to an E_α_ of approximately 104 kJ/mol, while the high-temperature peak corresponds to about 231.1 kJ/mol. In 15DP steel, the low-temperature peak corresponds to an E_α_ of about 110.2 kJ/mol, whereas the first and second high-temperature peaks correspond to 243.6 kJ/mol and 265.5 kJ/mol, respectively. Many studies [21,46,47] have attributed activation energies around 100 kJ/mol to reversible traps such as dislocations and martensite/ferrite (M/F) interfaces, while values around 230 kJ/mol are associated with carbides and precipitates, as well as more stable martensite/austenite (M/A) grain boundaries. In 15DP steel, the suppression of cementite precipitation and the refinement of grain boundaries result in significantly less hydrogen desorption at high-temperature peaks compared with 05DP steel. Moreover, a large amount of hydrogen is desorbed in 15DP steel at around 260 kJ/mol, which should be attributed to M/A interfaces. The increased Si content enhances the retained austenite fraction at room temperature, and M/A interfaces, acting as typical irreversible hydrogen traps, capture a considerable number of hydrogen atoms. It is also worth noting that a certain amount of ε-carbides exists in 05DP steel. As irreversible hydrogen traps, ε-carbides are considered to reduce HE susceptibility by lowering the diffusible hydrogen content [48]. Nevertheless, 05DP steel still exhibits high HE sensitivity, indicating that DP steels with multiphase structures are more vulnerable to hydrogen, and the underlying HE mechanisms require further investigation.

One viewpoint [2] holds that the high HE susceptibility of DP steel is attributed to the fact that hydrogen-induced cracks are prone to initiate along the ferrite–martensite phase interface. Firstly, although ferrite and martensite have the same crystal structure, the two phases tend to form a Cube-Cube orientation relationship and the corresponding {001}_α_ ‖ {001}_M_ habit planes. However, the difference in lattice constants will inevitably cause certain distortion energy and even mismatched dislocations near the phase boundary. The strain field thus provides the possibility for hydrogen enrichment at the interface. Secondly, the plastic deformation causes a higher density of movable dislocations in the ferrite near the two-phase interface in DP steel, which facilitates the migration of hydrogen along the dislocation movement, thereby increasing the hydrogen concentration in the martensite. In addition, the incompatibility of plastic strain between ferrite and martensite during deformation leads to stress concentration at the phase interface, further intensifying the enrichment of hydrogen at the interface and thus promoting the initiation of cracks.

In the microstructure of high-Si 15DP steel, M/A islands, blocky austenite, and retained austenite between the martensitic laths continuously experience stress. With increasing stress, martensitic transformation causes additional volumetric expansion, which aggravates the stress concentration tendency at phase boundaries. The martensite generated by the TRIP effect, as well as the fresh martensite obtained after over-aging and quenching treatment, makes the steel highly sensitive to hydrogen (increased high-binding-energy M/A interface traps, rapid fractographic transition from dimples to quasi-cleavage, and sharply increased elongation loss at high hydrogen charging levels). Hydrogen diffusing into the ferritic matrix migrates along M/F interfaces under stress, while hydrogen contained in retained austenite is released through martensitic transformation, and subsequently becomes trapped and concentrated at the M/A interfaces. The presence of hydrogen either reduces the cohesive energy of the lattice, alters the slip characteristics of the lattice, or ultimately leads to cleavage fracture in DP steel [49]. This may explain why, despite 15DP steel containing more retained austenite than 05DP steel, its susceptibility to HE remains relatively high.

## 5. Conclusions

This study systematically investigated the effects of silicon content and continuous annealing process parameters on the microstructure, mechanical properties, and HE susceptibility of DP 1500 steel. The main findings are summarized as follows:

1. By designing an appropriate continuous annealing process, DP steel with a tensile strength greater than 1500 MPa and an elongation close to 15% can be obtained, which possesses excellent mechanical properties.
2. The increase in Si content purify the ferrite matrix, inhibit the precipitation of carbides, enrich carbon in the austenite, increase the content of ferrite and retained austenite in the microstructure, and utilize the TRIP effect to further enhance the strength and plasticity of 15DP steel.
3. Although the increase in Si content enhanced carbon enrichment in austenite and refined the ferrite–martensite structure, thereby significantly improving strength and ductility, the fresh martensite formed either by strain-induced TRIP during deformation or after over-aging exhibited relatively high hydrogen embrittlement susceptibility. Consequently, despite the higher fraction and improved stability of retained austenite, 15DP steel did not exhibit substantially better HE resistance than 05DP steel.

## Figures and Tables

**Table 1:** Chemical composition of two experimental steels (wt. %).

Element	C	Si	Mn	P	S	Al	N
05DP	0.22	0.53	2.44	0.006	0.002	0.036	0.004
15DP	0.22	1.51	2.42	0.006	0.002	0.037	0.004

**Table 2:** Phase transition points of the two experimental steels.

Steel	A_c1_	A_c3_	M_s_	M_f_
05DP	738	920	402	212
15DP	751	947	390	198

**Table 3:** Mechanical properties of 05DP sample and 15DP sample, cathodically hydrogen charged at different charging times and current densities.

	Hydrogen Charging Current Density (mA/cm^2^)	Hydrogen Charging Time (min)	R_m_ (MPa)	R_p0.2_ (MPa)	A (%)	El_loss_ (%)
05DP	0	0	1434.93 ± 18.7	1043.6 ± 12.3	12.06 ± 1.54	0
	1	1	1215.87 ± 26.4	794.6 ± 18.9	7.79 ± 1.71	35.4%
	2	1	1132.95 ± 33.5	645.8 ± 24.1	5.22 ± 1.86	54.2%
	15	1	942.45 ± 41.6	650.5 ± 29.8	3.22 ± 1.93	73.3%
	2	5	845.72 ± 47.2	508.8 ± 33.5	3.87 ± 1.05	67.9%
	2	30	538.60 ± 52.9	480.2 ± 38.1	1.63 ± 1.14	86.5%
15DP	0	0	1530.41 ± 17.5	1055.5 ± 11.6	14.24 ± 1.57	0
	1	1	1274.48 ± 28.4	836.8 ± 20.4	11.45 ± 1.69	19.5%
	2	1	1254.38 ± 35.9	808.7 ± 26.7	8.89 ± 1.92	37.5%
	15	1	1037.22 ± 43.1	722.5 ± 31.4	4.94 ± 1.02	65.3%
	2	5	897.03 ± 46.5	662.4 ± 35.9	3.39 ± 1.11	76.2%
	2	30	607.75 ± 51.7	507.3 ± 39.6	2.89 ± 1.23	79.7%

**Table 4:** The hydrogen permeation test parameters of 05DP and 15DP steels.

Hydrogen Permeation Experiments	05DP Steel	15DP Steel
*L* (cm)	0.048	0.046
*I*∞L (A/cm^2^)	1.93 × 10^−6^	2.68 × 10^−6^
*t_L_* (s)	1330	850
*J*∞*L* (mol cm^−1^s^−1^)	1.24 × 10^−12^	8.53 × 10^−13^
*D_eff_* (cm^2^ s^−1^)	2.87 × 10^−7^	1.14 × 10^−7^
*C_app_* (mol cm^−3^)	4.32 × 10^−6^	7.48 × 10^−6^
*N_T_* (cm^−3^)	3.86 × 10^20^	1.68 × 10^21^

## Data Availability

The original contributions presented in this study are included in the article. Further inquiries can be directed to the corresponding author.
